# Assessment of Knowledge, Attitude, and Adherence to National Guidelines for Preventing Central Line-Associated Bloodstream Infections Among ICU Nurses of Adult Patients in Jeddah, Saudi Arabia: A Cross-Sectional Survey

**DOI:** 10.7759/cureus.42304

**Published:** 2023-07-22

**Authors:** Abdulrahim I Almalki, Hani A Alghamdi, Nidal A Tashkandy

**Affiliations:** 1 Preventive Medicine Postgraduate Program, Ministry of Health, Jeddah, SAU; 2 Preventive Medicine Department, Ministry of Health, Jeddah, SAU; 3 Infection Prevention and Control, Public Health Department, Directorate of Health Affairs, Ministry of Health, Jeddah, SAU

**Keywords:** healthcare-associated infection, infection control guidelines, infection control and prevention, central line-associated bloodstream infections (clabsi), central venous catheter infection, icu nursing, adult intensive care unit, jeddah saudi arbia

## Abstract

Background: Central line-associated bloodstream infections (CLABSIs) pose a significant burden on patient outcomes in intensive care units (ICUs). Adherence to evidence-based guidelines for CLABSI prevention is crucial in reducing healthcare-associated infections. This study aimed to assess the knowledge, attitude, and practice adherence to national guidelines for preventing CLABSIs among adult ICU nurses in Ministry of Health (MOH) hospitals in Jeddah, Saudi Arabia.

Methods: This cross-sectional survey included all adult ICU nurses with a minimum of one year of experience from the four major MOH hospitals in Jeddah with operational adult ICUs. A self-administered online questionnaire was utilized for data collection. Descriptive statistics, t-tests, ANOVA, and Pearson correlation were employed for data analysis.

Results: A total of 203 nurses completed the questionnaire (response rate: 91.5%). The overall knowledge score was 71%. Only 20% of nurses answered over 90% of the knowledge questions correctly, and merely 8% answered all questions correctly. Higher knowledge levels were significantly associated with older age, longer ICU nursing experience, higher education, holding a head nurse position, and attending educational courses on CLABSI prevention. Regarding attitudes, 58% of respondents had a positive perception of guideline utility for CLABSI prevention. In terms of adherence, the overall score was 65%, with only 5% reporting complete adherence to evidence-based practices for preventing CLABSIs.

Conclusion: This study highlights knowledge gaps, suboptimal adherence, and the need for targeted interventions to enhance nurses' understanding of and adherence to evidence-based guidelines for preventing CLABSIs among adult ICU nurses in Jeddah's MOH hospitals. Enhancing knowledge, attitudes, and practice adherence is crucial for reducing CLABSI risks and improving patient outcomes. Further research investigating the factors influencing nurses' knowledge, acceptance, and application of evidence-based guidelines is warranted to inform the development of tailored interventions and educational strategies.

## Introduction

Central line-associated bloodstream infections (CLABSIs) are a common and avoidable complication of central venous catheters (CVC) ​[1]​. CLABSIs lead to significant morbidity, mortality, prolonged hospital stays, and increased healthcare costs ​[2,3]​. Patients with CLABSIs are at a higher risk of in-hospital mortality and readmission compared to those without CLABSIs ​[4]​. The average cost of treating a single case of CLABSI is approximately $46,000 ​[5]​. 

A prospective surveillance study conducted in 109 Ministry of Health (MOH) hospitals in Saudi Arabia reported an overall CLABSI rate of 3.24 per 1,000 central line-days, which is higher than rates observed in developed countries ​[6]​. For example, in the United States (US), the intensive care unit (ICU) CLABSI rate is estimated to be 0.8 per 1,000 central line-days ​[1]​. These findings emphasize the need to address CLABSI prevention strategies in Saudi Arabia and implement effective measures aligned with international standards, following evidence-based guidelines for CVC insertion and maintenance. 

ICU nurses play a crucial role in providing quality care and ensuring patient safety in the ICU setting [7]. However, studies from various countries have revealed a lack of knowledge and adherence to best practices among ICU nurses, as outlined by guidelines from agencies like the US CDC ​[8-11]​. 

Assessing the knowledge and adherence of nursing staff to national guidelines for CLABSI prevention is crucial for identifying areas of improvement. Targeted educational interventions can then be implemented to enhance adherence, reduce the incidence of CLABSI, and improve patient outcomes. Limited research exists on this issue in Saudi Arabia, particularly concerning ICU nursing staff in MOH hospitals in Jeddah. Therefore, this study aimed to assess the knowledge, attitude, and practice adherence of adult ICU nursing staff in MOH hospitals in Jeddah, regarding national evidence-based guidelines for preventing CLABSIs. 

## Materials and methods

Study design and study population

This cross-sectional survey was conducted between April and June 2023. All ICU nurses (n=222) working in adult ICUs for at least one year in the major four MOH hospitals in Jeddah with operational adult ICUs were invited to participate. The inclusion criteria were selected based on the experience level of the nurses to ensure an adequate understanding of CLABSI prevention guidelines. 

Data collection

Data was collected using an online self-administered questionnaire based on Saudi MOH guideline recommendations for the prevention of CLABSIs, which are identical to the CDC guidelines. The questionnaire, originally designed and validated by Luciana Albano et al., underwent a modification in which all references to the CDC guidelines were changed to the Saudi MOH guidelines [12]​. This change was made to ensure alignment with the guidelines familiar to the nursing staff in the participating hospitals, as they commonly refer to the Saudi MOH guidelines instead of the CDC guidelines. 

The questionnaire consisted of five sections: demographic and professional data, knowledge of healthcare-associated infections and CLABSI prevention based on the Saudi MOH guidelines, attitudes toward the utility of guidelines, behaviors and practices related to CVC insertion and management, and sources of information and educational needs. The knowledge assessment section comprised 11 multiple-choice questions (MCQs) related to knowledge about the CDC's main recommendations for preventing CLABSI. The questionnaire demonstrated a high degree of internal consistency, as evidenced by a Cronbach's α value of 0.83 and an inter-item correlation coefficient (r) of 0.46 ​[12]​. 

Statistical analysis

Continuous variables were summarized using mean, standard deviation, or range (for age), while categorical data were presented as numbers and percentages. The knowledge score for each respondent was calculated by assigning one point for every correct answer. These scores were then summed up to represent the total number of correct answers out of a maximum score of 11. The overall knowledge score for the knowledge section was reported as a percentage, representing the proportion of correct answers achieved out of the total possible correct answers for all respondents. Adherence and attitude rates were presented as percentages. Total scores between two groups were compared using an independent t-test, and for comparisons among three or more groups, a one-way ANOVA was conducted followed by Bonferroni post-hoc pairwise comparisons. Data analysis was performed using IBM SPSS Statistics for Windows, Version 24.0 (IBM Corp., Armonk, NY). A two-tailed p-value of less than 0.05 was considered statistically significant. 

Ethical considerations

Ethical approval was obtained from the Ministry of Health Institutional Review Board in Jeddah (code A01604) prior to data collection. Informed consent was obtained from all participants, and confidentiality of data was ensured throughout the study. 

## Results

A total of 203 nurses completed the questionnaire (response rate: 91.5%). Among them, 164 (80.8%) were female, with a mean age of 33 (range: 23-54). The majority held a bachelor's degree (78.3%, n=159), and a significant portion had attended educational courses on CVC maintenance (79.3%, n=161). Details of participants' characteristics are presented in Table 1. 

**Table 1:** Characteristics of the respondents (N=203). ^a ^Age reported as mean and range ICU, intensive care unit; MOH, Ministry of Health; CVC, central venous catheter

Characteristic	Frequency (%)
Gender	
Male	39 (19.2%)
Female	164 (80.8%)
Age, years^a^	33 (23-54)
Highest educational level	
Diploma	37 (18.2%)
Bachelor's degree	159 (78.3%)
Master's degree	7 (3.4%)
Nursing level	
Nurse	189 (93.1%)
Head Nurse	14 (6.9%)
Length of ICU nursing experience, years	
1 – <3	39 (19.2%)
3 – 5	32 (15.7%)
6 – 10	78 (38.4%)
> 10	54 (26.6%)
Have you ever attended educational courses on the maintenance of CVCs?	
Yes	161 (79.3%)
No	42 (20.7%)
Is there an internal care protocol in your hospital for the prevention of intravascular catheter-related infections?	
Yes	195 (96.1%)
No	8 (3.9%)
Do you follow the Saudi MOH Guidelines for the prevention of intravascular catheter-related infections as a source of information?	
Yes	200 (98.5%)
No	3 (1.5%)
Do you feel that you need more information regarding the prevention of CVC-related infections?	
Yes	133 (65.5%)
No	70 (34.5%)

**Figure 1:**
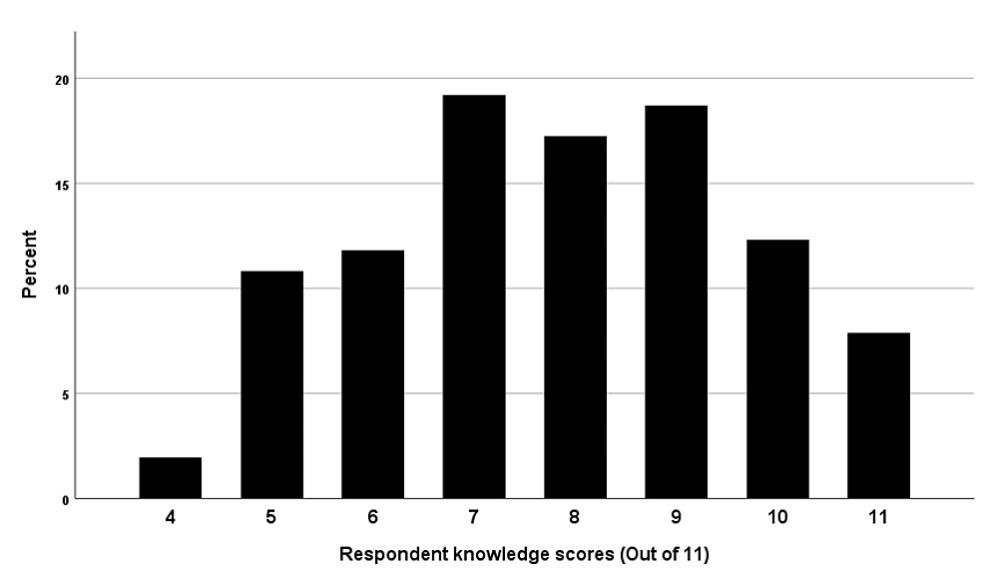
Distribution of knowledge of national guideline recommendations for preventing CLABSI scores among respondents. CLABSI, central line-associated bloodstream infections

The knowledge section of the questionnaire had an overall correct response rate of 71%. The mean score for the 11 questions in this section was 7.84, with a standard deviation of 1.82. Scores ranged from 4 to 11. Only 20% (n=41) of participants answered more than 90% (10 or more questions) correctly, while a mere 7.9% (n=16) answered all questions correctly. The distribution of knowledge scores is presented in Figure 1. 

Table 2 provides a breakdown of the questions in the knowledge of guideline recommendations for preventing CLABSI section of the questionnaire, along with the corresponding correct response rates. 

**Table 2:** Responses to the "Knowledge of national guideline recommendations for preventing CLABSI" section of the questionnaire. CVC, central venous catheters; CLABSI, central line-associated bloodstream infections

Question	Number of correct responses	Percentage of correct responses
The routine replacement of CVCs is a recommended strategy to prevent infection?	74	36.50%
The use of antibiotic ointment at the CVC insertion site is recommended for reducing infections	112	55.20%
How often should administration sets used for standard infusions be replaced?	117	57.60%
How often should a clean and intact sterile gauze on the catheter insertion site be replaced?	117	57.60%
If the adherence to aseptic technique cannot be ensured during the CVC insertion, the catheter should be replaced within 48 hours	132	65.00%
How often should a clean and intact transparent dressing on the catheter insertion site be replaced?	139	68.50%
What is recommended to be used for cleaning the skin before CVC insertion and during dressing changes?	152	74.90%
Have you ever attended educational courses about the maintenance of CVCs?	161	79.30%
How often should the administration sets used to administer blood, blood products, or fat emulsions be replaced?	186	91.60%
Insertion and maintenance of CVCs must be preceded by hand hygiene procedures	199	98.00%
Insertion of CVCs must be performed using sterile gloves	202	99.50%

Higher levels of knowledge were found to be significantly associated with age (Pearson’s r=0.5), higher education (master's degree), holding the position of a head nurse, having a longer length of ICU nursing experience, and attending educational courses on CVC maintenance. Table 3 presents a summary of the results from subgroup analyses, which were stratified by demographic and other variables. 

**Table 3:** Subgroup analysis of knowledge scores. Post-hoc comparisons utilized the Bonferroni test following significant one-way ANOVA results ICU, intensive care unit; CVC, central venous catheter; SD, standard deviation

Characteristics	Mean±SD	Range	Significantly different from group	t/F	P-value
Total cohort (N=302)	7.84±1.82	4 – 11			
Gender				0.216	0.83
Male (n=39)	7.9±1.9	4 – 11	–		
Female (n=164)	7.8±1.8	4 – 11	–		
Highest educational level				10	< 0.001
Diploma (n=37)	8.27±1.5	4 – 11	C		
Bachelor's degree (n=159)	7.62±1.8	5 – 11	C		
Master's degree (n=7)	10±0.78	9 – 11	A, B		
Nursing level				-12.68	< 0.001
Nurse (n=189 )	7.6±1.7	4 – 11	Head nurse		
Head nurse (n=14 )	10.2±0.61	9 – 11	Nurse		
Length of ICU nursing experience, years				30.49	< 0.001
A. 1 – <3 (n=39 )	6.1±1.3	4 – 9	B, C, D		
B. 3 – 5 (n=32 )	7.5±1.5	5 – 10	A, D		
C. 6 – 10 (n=78 )	7.95±1.5	5 – 11	A, D		
D. >10 (n=54 )	9.13±1.5	5 – 11	A, C, D		
Attendance of educational courses on CVC maintenance				7.15	<0.001
Attended courses (n=161 )	8.2±1.7	4 – 11	Never attended courses		
Never attended courses (n=42 )	6.3±1.4	4 – 9	Attended courses		

The overall correct response rate regarding the utility of guideline recommendations in preventing CLABSI was 65%. Only 58% of respondents perceived the utility of guidelines positively for CLABSI prevention. 

In terms of adherence to recommended evidence-based practices for CVC maintenance, the overall rate of correct responses was 65.5%. However, it is important to note that none of the participants reported performing CVC insertion, as this procedure is almost always conducted by a physician in MOH hospitals. Therefore, no responses related to the practice adherence to CVC insertion were received. Only a small 5% of respondents reported complete adherence, scoring a maximum of 5 out of 5, to guideline recommendations in other aspects of CVC maintenance. 

Table 4 provides a breakdown of the questions in the "adherence to recommended evidence-based practices for CVC maintenance" section of the questionnaire, along with the corresponding correct response rates. 

**Table 4:** Responses to the "Adherence to recommended evidence-based practices for CVC maintenance" section of the questionnaire. CVC; central venous catheters

Question	Number of correct responses	Percentage of correct responses
Before replacing catheter site dressing, do you perform hand hygiene?	203	100.00%
How often do you visually monitor the catheter site when changing the dressing?	178	87.70%
How often do you perform the palpation of the site through the intact dressing on a regular basis?	105	51.70%
How often do you remove the site dressing if patients have tenderness to allow a thorough examination of the site?	157	77.30%
If a patient with a central line catheter has a fever, what is the best next step?	21	10.30%

Knowledge scores showed a weak positive correlation with the perception of guideline utility for CLABSI prevention (r=0.256) and practice adherence scores (r=0.147). 

## Discussion

This study included adult ICU nurses from the four major MOH hospitals in Jeddah city with operational adult ICUs. Our findings show a moderate overall knowledge level of CDC guideline recommendations, with an average score of 71%. Notably, only 20% of participants answered over 90% of the questions correctly, and just 7.8% answered all questions correctly. Compared to the study conducted by Almahmoud et al. at King Abdul-Aziz Medical City in Riyadh, Saudi Arabia, our participants demonstrated a lower knowledge level (82% in Riyadh) ​[13]​. It is important to consider that their study used a different questionnaire and was conducted in a specialized tertiary medical center. Interestingly, the knowledge level in our study is higher than the average reported in similar studies from various countries ​[9,14-17]​. 

Multiple questions in the CDC guideline recommendations knowledge section of the questionnaire received remarkably low rates of correct responses, indicating notable knowledge gaps. For instance, a mere 36.5% of nurses were aware that routine replacement of CVC is not recommended for infection prevention. Similarly, only 55.2% of participants knew that using antibiotic ointment at the CVC insertion site is not recommended. Additionally, just 57% accurately identified the proper timing for replacing administration sets and clean, sterile gauze at the insertion site. 

Older age, having a longer length of ICU nursing experience, higher education (master's degree), holding the position of a head nurse, and attending educational courses on CVC maintenance were significantly associated with higher levels of knowledge scores. These results are consistent with prior research, highlighting that increased nursing experience, along with formal education and training, can enhance familiarity with evidence-based guidelines for preventing CLABSIs ​[9,16-18]​. 

Only 58% of the respondents had a positive perception of the guidelines' utility in preventing CLABSI, indicating a considerable proportion with unfavorable views on their effectiveness. This highlights a notable gap between knowledge and perception among the participants, underscoring the importance of exploring factors influencing healthcare professionals' acceptance of guidelines. Investigating these factors is crucial to ensure successful implementation and adherence to CLABSI prevention guidelines. 

The study found an overall adherence rate of 65.5% to evidence-based practices for CVC maintenance. However, only 5% of respondents reported complete adherence, scoring a maximum of 5 out of 5, highlighting a significant gap between knowledge and practice. Ongoing education and targeted interventions are needed to improve adherence among nursing staff, minimize CVC-related infection risks, and enhance patient outcomes. Further research should explore factors influencing adherence to guide the development of effective interventions and educational strategies. 

Limitations of this study include its limited generalizability to other regions or practices beyond MOH hospitals in Jeddah. Additionally, focusing solely on ICU nurses may not capture the full spectrum of CVC procedures in various departments. The reliance on self-reporting introduces potential bias, and the unsupervised nature of data collection raises concerns about participants looking up answers. Furthermore, the cross-sectional design restricts causal interpretations. Future research should address these limitations by considering diverse settings, employing objective assessment methods, and utilizing longitudinal designs. 

## Conclusions

In summary, this study highlights knowledge gaps, negative perceptions, and inadequate adherence to evidence-based guidelines for preventing CLABSIs among ICU nurses in Jeddah's MOH hospitals. To address these issues and enhance patient safety, targeted educational interventions, continuous training programs, and regular guideline updates are recommended. Further research is needed to understand the factors influencing nurses' acceptance and application of the guidelines, facilitating the development of effective strategies for improving adherence and promoting infection prevention practices in the ICU. 
